# Visual-Based Defect Detection and Classification Approaches for Industrial Applications—A SURVEY

**DOI:** 10.3390/s20051459

**Published:** 2020-03-06

**Authors:** Tamás Czimmermann, Gastone Ciuti, Mario Milazzo, Marcello Chiurazzi, Stefano Roccella, Calogero Maria Oddo, Paolo Dario

**Affiliations:** The BioRobotics Institute of Scuola Superiore Sant’Anna and Department of Excellence in Robotics and AI of Scuola Superiore Sant’Anna, 56025 Pontedera (PISA), Italy; gastone.ciuti@santannapisa.it (G.C.); m.chiurazzi@santannapisa.it (M.C.); stefano.roccella@santannapisa.it (S.R.); paolo.dario@santannapisa.it (P.D.)

**Keywords:** defect detection, classification, deep learning, industry 4.0, survey

## Abstract

This paper reviews automated visual-based defect detection approaches applicable to various materials, such as metals, ceramics and textiles. In the first part of the paper, we present a general taxonomy of the different defects that fall in two classes: visible (e.g., scratches, shape error, etc.) and palpable (e.g., crack, bump, etc.) defects. Then, we describe artificial visual processing techniques that are aimed at understanding of the captured scenery in a mathematical/logical way. We continue with a survey of textural defect detection based on statistical, structural and other approaches. Finally, we report the state of the art for approaching the detection and classification of defects through supervised and non-supervised classifiers and deep learning.

## 1. Introduction

Defect detection and classification are two topics that need to be treated as unique problems related to the field of artificial vision. Digital image processing problems mainly derive from specific conditions in which researchers aim to mimic or substitute human vision and decision methodologies with artificial techniques. The general purpose of mimicking human vision is to identify and classify a subject: these two goals are always strictly bonded together. Literature on artificial visual processing is usually categorized into visual processing algorithms, which consist in the recreations of the human vision, and classifiers, which are a remodeling of the human decision techniques. In this paper, we address both categories, but, instead of summarizing all the visual processing methodologies, we focus on the specific solutions that are strongly related to visual processing methods and, specifically, on visual inspection techniques for metallic, ceramic or textile surfaces in industrial applications.

**Figure 1:**
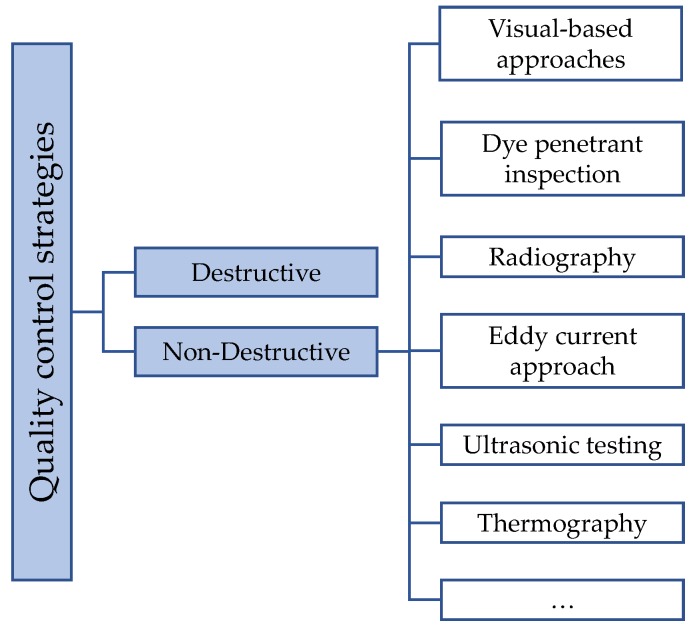
Categorization of the quality control strategies.

Quality control is a crucial aspect in the industrial production line. Several approaches are currently used to assess the quality of a product or the outcome of a process. Depending on the method employed to identify a defect on a surface/volume, quality control strategies can be classified as destructive or non-destructive, as shown in Figure 1. Non-destructive testings (NDTs) aim at monitoring a component to detect a defect without extracting samples from it, or permanently damaging it. Mostly used in the aeronautic field, NDTs are classified as: visual-based method, dye penetrant inspection, radiography, ultrasonic testing, eddy current approach, and thermography [1].

Among them, the visual-based approach for defect detection is one of the most common procedures in industry. However, the traditional visual inspection is a non-measurable process with variable and subjective outcomes. This has pushed researchers to develop new automatic defect detection systems with demanding requirements because of the complexity and uniqueness of any specific problem to solve. However, such a system depends on the material properties of the surfaces to monitor and on the environmental conditions. In industrial applications, indeed, the environment complicates any implementation, due to dusty or resonating working areas.

The description of a defect and its categorization is a procedure that involves series of subjective decisions. The main attributes of a defect depend on the aimed precision and resolution of the detection procedure, since the size of defects can differ among industrial applications. It is highly advised to establish a quality standard of the product in every industrial quality control application before designing and implementing the automatic system.

This review is organized as follows. in Section 2, we propose a taxonomy of defects that can occur on metal surfaces, which is based on the relevant materials’ properties and objects industrial categorization for quality inspection. In Section 3, we describe the representation and processing of the defects through images. In Section 4, we propose a supplementary list of detection methods for defects and detail them, based on their methodology and efficiency. In Section 5, we describe the supervised and unsupervised classification methods used for image processing.

## 2. Taxonomy of Surface Defects

In the industrial production area, quality control aims at maintaining a quality level or at localizing the defects for further repair. Conventional detection methods usually deal with regular, macro-sized and complex variations of surface defects. Almost every artificial visual defect detection technique aims to detect imperfections and classify them for further processing.

**Figure 2:**
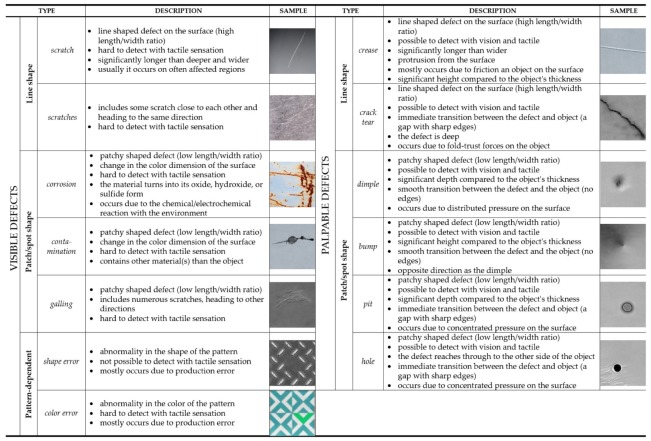
The category of visible defects contains defects that are hard or almost impossible to be localized by touch. The category of palpable defects contains defects that are significantly easier to be localized with the combination of vision and touch. Clearly, the naming of categories does not mean that there are no exceptions in either category.

For a proper classification, industrial applications need well-structured databases of the possible defect types. However, establishing such a general and comprehensive database for a classifier is challenging, due to the randomness and uniqueness of the defects that can occur in the operation scenarios. In this regard, although a general categorization approach is highly demanded, almost every application employs a material-based defect classifier. Observing the referred papers in this study and gathering examples from them, we propose a low-level, unified categorization for defect types, as reported in Figure 2. This taxonomy of surface defects, applicable to any material, is classified into two major groups: visible and palpable defects. It is worth noting that this categorization is still basic, essentially conceptual and not satisfactory for procedures with specific requirements, but it provides a strong and reliable basis for a classification with artificial intelligence system. The fundamental assumption of this defect categorization is that the classification of a defect is a severely subjective judgement, i.e., it greatly depends on what a defect represents for the human supervisor. This decision is usually based on a threshold and a logical-based representation of the size ratio of both the component and the defect. Therefore, the structure of the taxonomy is mainly organized by size ratios and spatial features.

## 3. Artificial Visual Processing Techniques

**Figure 3:**
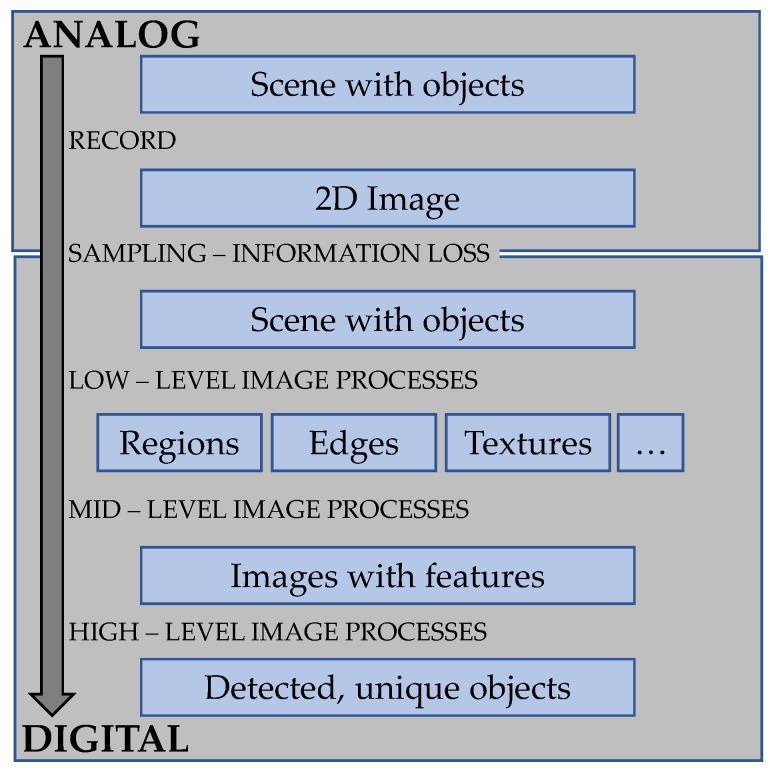
The theoretical levels of image representation for image analysis. The transformation from the analog to the digital domain (with sampling) always results in some information loss.

The main goal of visual-based approaches is to understand the world both in its natural and artificial representations. In the latter, the process to identify images is mostly an attempt to look for a mathematical/logical connection between the input images and representations of the environment. The mathematical/logical connection is a transition from the input image (s) to the model, which reduces the information contained in the image to relevant information for the application domain. Image representation can be roughly divided into four levels, as described in Figure 3. The hierarchy of image representation and the background functions/algorithms can be further simplified as low-level and high-level image processing.

Most low-level processing methods do not use any prior knowledge about the content of the image. This means that the methods that belong to this group can be applied to every image without considering any information about the prerecorded environment. This group includes: (1) image compression; (2) pre-processing; (3) sharpening; and (4) edge extraction methods.

The higher-level processing methods are more complex and operate above the mathematical representation of the image (e.g., in the content domain, where unique objects or extra information have been already described) by establishing classifiers and where mimicking the human cognition is needed.

As depicted in Figure 3, to reach the level of the “images with features”, and to have a picture with content, several attributes of the image—e.g., edges, textures, etc.—have to be described. Two distinct principles apply for naturally occurring visual observations. One is performed according to previous knowledge about the object to be found. The second is performed with no given information about the object, but with knowledge on the environment, considered as normal. Usually, most non-destructive visual inspection methods to find surface abnormalities involve textures and/or color analysis, performed by low-level processes. These principles can be replicated in artificial systems, but employing different approaches. To recognize individual defects on a surface, a descriptor database of the possible defects, such as a classifier, must be established. Although they have to deal with false positive (FP) and false negative (FN) outcomes, classifiers, can be used to ensure that the system is able to recognize a defect. Modeling the second principle leads to texture analysis problems, where any deviation from the normal pattern in the texture is recognized and highlighted.

## 4. Textural Defect Detection

Textures provide important and unique information for artificial visual detection and identification systems. The latter use different types of texture analysis and classifications because the general task of defect detection is mostly a texture analysis problem. The most promising and accurate approach to describe a texture is to extract its unique features, although this turns out to be a challenging task.

Xie et al. [2] categorized the techniques for extracting texture features into four categories with references and their comparison (based on the work of Luo et al. [3]) to distinct papers in this field (see Table 1 and Table 2), and then Ngan et al. [4] in 2011 made a new extended review.

The statistical and filter-based approaches have been the most popular approaches used to date. The categorization, provided in [2], is a well-structured analysis and it can be used as a starting point for future summarizations. The four main categories, namely statistical approach, structural approach, filter-based approach, and model-based approach, could be discussed in the same group, but it is advisable to discuss them separately from the color texture analyzing techniques. The main differences between the two groups are the color related features. Thus, Xie et al. [2] focused only on non-color related methods.

Based on several research studies in the field of visual inspection (e.g., [5]), it is possible to use the best fitting approach for a specific problem. It is worth mentioning that providing an exhaustive survey of all texture features is not practical or ie even impossible due to their enormous diversity. All different approaches can be evaluated according to the following features.

### 4.1. Statistical Approaches

Statistical approaches focus on analyzing the spatial distribution of pixel values in a recorded image. In this category, it is possible to count numerous publications and techniques, ranging from low-level to higher-order statistics, such as histogram statistics, autocorrelation, local binary patterns (LBP) and others (see Table 2).

Histogram properties and statistics are useful supports for both higher-level and low-level processes with low computational cost. These processes include operations from statistics, such as sensitivity range, mean, geometric mean, harmonic mean, standard deviation, variance and median. It also includes other histogram comparison statistics used for texture features, such as L1 norm, L2 norm, Mallows or EMD distance, Bhattacharyya distance, Matusita distance, divergence, histogram intersection, Chi-square and the normalized correlation coefficient. Implementations can be found, for instance, in [6,7,143,144,145], which have proven to be worthy as low-level processes in defect detection. They can be also used as tonality disruptors (e.g., [144,146]) due to their properties of being invariant to translation and rotation, and being insensitive to the exact spatial distribution of color pixels. Yuan et al. [8] proposed an improved Otsu thresholding method, called Weighted Object Variance (WOV), and achieved 94% of detection rate with 8.4% of false alarm rate. In another recent study, the authors also proposed and improved Otsu thresholding method, but without reporting detailed results on the method’s performance [9]. Recently, Dastoorian et al. [10] implemented an Adaptive Generalized Likelihood Ratio (AGLR) approach in a practical case study to inspect 27 samples, each with a unique fault using 3D laser scanning technology. The method detects defects by checking whether the distribution of the observed data is significantly different from a baseline historical distribution in an adaptive manner.

- $Sensitivity=\frac{TP}{TP+FN}$
- $Specificity=\frac{TN}{TN+FP}$
- $Detection\;Success\;Rate=\frac{TP+TN}{TP+FN+TN+FP}$

where TP, TN, FN and FP stand for true positive, true negative, false negative and false positive, respectively.

Spatial grey level co-occurrence matrices (GLCMs) are one of the most well-known and commonly used texture features [147]. These are statistical methods that measure the spatial relationship of grey-scale pixels into co-occurrence matrices. The GLCM functions characterize the texture of an image by calculating how often pairs of pixels with specific values and in a specified spatial relationship occur in an image, given a displacement vector, and then extract texture features such as energy, entropy, contrast, homogeneity and correlation from these matrices. There are several publications describing applications using co-occurrence matrices to detect defects [6,11,15,16,17,18,19,20]. Despite the high number of applications, the co-occurrence matrix features have several deficiencies. Several reports [33] have proved that there is no generally accepted optimization for the displacement vector, and GLCMs have other statistical and computational dependencies. In other comparative studies [16,17], the co-occurrence matrices have shown worse performance in defect detection compared to the Markov Random Fields (MRF), filtering-based, LBP methods. In contrast to other papers, such as [20], the authors showed the opposite result compared to Gabor filter based approach, with a slightly better performance. Recently, the GLCM method has become a very popular approach: Capizzi et al. [29] proposed an GLCM based detector with a radial basis probabilistic neural network to detect defects on fruits, and achieved 97.25% detection rate with 2.75% false alarm. In [21], the authors used the GLCM and other methods to extract features to train a supervised Support Vector Machine (SVM). In [148,149], the authors achieved 93.4% and 83.3% defect detection rate to replace manual defect inspection, respectively. In [22], the authors used the GLCM for textural feature description and combined it with a Fast Discrete Curvelet Transform (FDCT), achieving 93.3% detection rate with 3.6% false alarm on ferrite magnetic tiles.

The methods using autocorrelation can be applied on textures that contain repetitive patterns, such as textiles. This mathematical algorithm aims at finding correlation between the texture and its translation with a displacement vector and derives vertexes in case of high regularity. However, this type of method fits for several defect detection problems, due to the high sensitivity to noise interference and the only application capability on patterned textures makes it unsuitable for most of the detection tasks because of their random surface. Zhu et al. [23] published a study about a yarn-dyed fabric defect detector, which combines autocorrelation with the GLCM; however, the system was tested only on 16 samples and no detailed results were reported.

Local binary pattern (LBP) is a computational low cost and very efficient texture operator. It calculates thresholds of the grey-scale pixels and its neighbors in a sliding window, which uses the center pixel of the window as a threshold. It considers the threshold’s result as a binary number. It was first published by Ojala et al. [32] as a labeling visual descriptor for textures. The LBP operator is insensitive to changes in illumination and image rotation, and it makes it a robust operator. It has similarities in the logic of computation with co-occurrence matrices, but the LBP seems to achieve lower performance [33]. It has been used in several defect detector applications on varied materials [12,33,34,35,150] such as ceramics or wood. Recently, Zhang et al. [30] combined the GLCM and LBP methods to extract image features to train a BP Neural Network: it achieved 97.6% detection rate on 90 samples from TILDA database [151]. In [38], Sindagi and Srivastava proposed a modified LBP method to train a SVM classifier, detecting defects with 93% accuracy on 148,905 samples.

### 4.2. Structural Approaches

Structural Approaches (SA) mostly focus on the spatial location of the texture elements. These elements can be extracted from the texture and described as texture primitives. Applying spatial arrangement rules to texture primitives can result in a dynamic texture model. The texture primitives are mostly simple grey-scale regions, line segments or individual pixels. These elements are always used in a combination with placement rules which are derived from the geometric relationships or spatial statistics of these primitives. After revising several publications about SAs (see Table 2), it is possible to state that this approach performs much better on patterned regular textures.

Several methods for SAs have been developed, such as the one proposed by Chen and Jain [54] that describes a model with a skeleton structure of the texture or the one proposed by Bennamoun and Bodnarova [55] that describes an approach called texture blob detection: both approaches are made for defect detection on fabric images. Wen and Xia [53] examined the surface of leather extracting the edge segments and statistically evaluated them based on their physical attributes. Morphological operators are other SAs, developed by Matheron and Serra [152] in 1964. They give an outstanding opportunity for segmenting defects and general defects detection, as reported in [56]. Recently, Tolba and Raafat [57] proposed a multiscale structural similarity index (MS-SSIM)-based method, which discriminated abnormal features with a 99.1% success rate. Yun, Jong Pil et al. [60] proposed an automatic defect detection optical system based on morphological operations, using backlight technique. Finally, in [58], the authors developed a new method: the prior knowledge guided least squares regression (PG-LSR) and solved the subspace segmentation problem. However, no detailed results of the detection were reported.

### 4.3. Filter-Based Approaches

Images can be described by detected features, such as edges, textures and regions (Figure 3). Filtering these features is one of the earliest attempts in image processing, especially for the extraction of the edge details. It is also a low-level process and the edges can be interpreted as spatial impulsive intensity changes of the image [61]. To extract edges from images, it is possible to use several filters and algorithms in the spatial domain, such as Sobel, Robert, Canny, Deriche, Laws and Laplacian filters. Neubauer [62] introduced a method with three 5×5 finite impulse response (FIR) filters as first-order statistics, and performed defect segmentation with TP = 98.3% and TN = 90.6%. Unser and Ade [63,64], and then Monadjemi et al. [33,65], used texture independent ensemble of macro windows called eigenfilters as defect detectors. Eigenfilters are considered highly sensitive to local distortion and noise; it makes them less suitable for online fabric inspections. However, their low complexity and ability to incorporate various time- and frequency-domain constraints easily compared to other methods, have made eigenfilters very commonly used as general approach.

In most cases, operating in the spatial domain involves noise and complications to find a direct kernel. Therefore, transforming the images into the frequency domain with Fourier Transformation (FT) gives the leverage to easily filter the noise and process the image, as described in [71]. The basic logics are to transform the image into Fourier domain, filter and then re-transform it into the spatial domain. The differences between the original and processed images can be considered as potential defects, based on the applied function in the transformation [72]. Chan and Pang [73] applied a central spatial frequency spectrum, based on the idea that defects usually occur in horizontal and vertical directions. However, these filters suffer the assumption that textures are periodic. D’Astous and Jernigan [74] used peak and power distribution features to discriminate textures. Optical Fourier transform (OFT) has been used in several applications performed on fabrics, such as in [75] by Hoffer et al.; in [76] by Castellini et al.; in [77] by Ciamberlini et al.; and in [78,79] by Campbell et al. Recently, Shan Gai [80] performed the quaternion wavelet transform on 10,000 banknote images and achieved 97% defect detection success rate and 0.35% false alarm.

The Fourier transformation depends on the entire image because of its coefficients. This property makes it unable to localize defects in the spatial domain. The most common solution for this problem is to apply a windowed FT for spatial dependency and, if the window function is Gaussian, it results in the well-known Gabor transform. The Gabor transform (GT) attempts the optimal joint localization in spatial and spatial-frequency domains [153]. There are two types of approaches to this method. The first is when several filters have been stored in predetermined frequencies and orientations to cover all possibly occurring frequencies in the image and calculate their correlation [85]. However, this approach can be computationally intensive to achieve high recognition quality. The second approach concerns the implementation of the optimal filters that correlate with the desired recognition area in parameters, but achieving the optimal settings is hard and crucial [86]. Turner [87] and Clark et al. [88] first proposed the use of Gabor filters (GF) in texture analysis. In the past decades, several applications were published about Gabor filters [33,68,86,89,90,91,92,93,94]. Kumar and Pang [91] used only real Gabor functions for fabric defect detection, and then, in [68], they used similar features on plain and twill fabrics in three schemes with no explicit results in the first scheme, but with 100% accuracy in the second and third schemes. They also investigated the imaginary part of the Gabor functions as an edge detector. In [86,95], Bodnarova et al. applied a Fisher cost function to select a subset of Gabor functions to perform flaw detection on textiles and achieved 82.86% accuracy with the proposed optimal two-dimensional GF. Escofet et al. [89] performed a multi-scale and multiresolution Gabor filtering in a novelty detection framework. Among the GT methods, Mak and Peng [85] achieved the best detection results on a fair number and quality of samples. They achieved 96.2% success rate with a Gabor wavelet network to extract optimal texture features from a defect-free image and then 97.1% with an only real Gabor filter for defect detection. Recently, Kang et al. [39] proposed two approaches: an optimized Gabor filter and a distance-matching-based method called regular band. They achieved a 71.4% detection rate with 0% false alarm with Gabor filtering and a 93.1% detection rate with 4.9% false positives on 85 sample images from TILDA database with regular band. Hu [96] established an elliptical Gabor filter tuned by a simulated annealing algorithm, but no detailed defect detection results were published.

With similar properties to the Gabor transform, Wavelet Transform (WT) representations have also been used as defect detectors [25,97,98,99,100,101,102,103,104,105,106]. WTs are based on small waves of varying frequency and limited duration called wavelets and provide local information from horizontal, vertical and diagonal directions on any input image [107]. Several approaches managed to achieve 98–100% success rate for defect detection with the Fuzzy Wavelet Analysis [108], multiscale wavelet method [109], WT image restoration schemes [72,100] and adaptive level-selecting scheme to analyze co-occurrence matrices [111]. However, the reliability of these methods is questionable due to a limited dataset of samples used during the tests. In [99,112], Sari-Sarraf and Goddard performed discrete WTs and edge fusions to emphasize the defects from the background on fabric images. The procedure achieved an 89% detection success rate over 3700 images of fabrics, containing 26 distinct kinds of defects. Yang et al. [154] developed an adaptive wavelet-based feature extractor with a Euclidean distance-based detector for fabric images, which achieved 97.5% with a defect-database (480 defect-free and 480 defective samples), and 93.3% without a defect-database (780 defect-free and 180 defective samples). Later, in [113], they outperformed five other WT-based methods with their new Discriminative Feature Extraction (DFE) method, reaching 95.8% classification accuracy. In [114], Lin used one-level Harr wavelet transform to detect ripple defects on chips.

Recently, Zhou et al. [70] proposed two new saliency detection method—region growing geodesic saliency (RGGS) and region growing Euclidean saliency (RGES)—and template matching with multiscale mean filtering. Their template matching method achieved an 88.83% detection rate, while the RGES method performed with an accuracy of 75.95%.

### 4.4. Model-Based Approaches (MBAs)

Model-based methods are classified into three groups: (1) fractal models; (2) autoregressive models; and (3) random field models. Fractals play a significant role in the description of the natural surfaces with self-similar and irregular texture. They were firstly reported by Mandelbrot [117]. In [118,119], Conci and Proenca introduced a differential box-counting method with non-overlapping copies of images and achieved a 96% success rate on 80/75 defect-free/defective samples. Bu et al. [155] performed defect detection based on four fractal features and support vector data description on seven datasets of 14,378 defect free samples and 3222 defect samples with a 98.3% success rate. Kaneko [156] achieved 93.85% accuracy of classification on 65 samples of the Brodatz texture database [157]; however, the method is computationally heavier compared to the technique presented in [158]. In a comparative study by Ohanian and Dubes [120], the fractal method performed well against GLCMs, Gabor filters and MRF-based methods; however, its success heavily depends on the self-similarity of the texture and therefore it provides weaker performance.

Markov random fields approaches [121] combine both statistical and structural information of context dependent entities, such as pixels depending on their neighbor pixels, and can be used in texture segmentation [122,123] and classification problems [124,125]. Cohen et al. [126] used Gaussian MRF (GMRF) to model defect-free texture on fabric images. They treated the method as a hypothesis testing problem on the statistics derived from the GMRF model. Six 256×256 testing images with various defects were divided into non-overlapping sub-blocks, where each block was classified as defective or non-defective. Although the detection success rate was high, the reliability of the testing is questionable due to a limited dataset of samples. Özdemir and Ercil [127] compared their MRF-based method in fabric inspection and a Karhunen–Loeve (KL)-based method; then, in [26], the authors determined the competitiveness of the MRF model against other statistical and spectral based methods. In 2000, Baykut et al. [130] applied the aforementioned GMRF method in real-time application with a dedicated DSP system. In 2005, Chan et al. [128] proposed a wavelet-domain Hidden Markov Tree model with a level set segmentation technique. Recently, in [129], the authors described a defect pavement detection method, and improved a quality of image segmentation by Markov random fields and Graph cuts method with an unsupervised Random Forest learning methodology for classification. Moradi and Zayed [132] developed a real-time application for defect detection in sewer tunnels by using Hidden Markov Model (HMM) and achieved 82.5% detection rate on 40 samples.

The main concept of the autoregressive model (AR) is to characterize texture features based on the linear dependencies of pixels [24]. Serafim [134,135] applied multiresolution pyramids for leather defect segmentation of natural images based on two-dimensional AR models. Basu and Lin [136] used a multi-scale AR texture model on tress for fabric samples, while, in [137], the authors used one-dimensional AR and a CCD camera for a real-time web inspection. From these studies, it is possible to state that lightning is a crucial component of the inspections. Although the testing outcomes were very promising, no quantitative results were published at the end of the tests [156]. Recently, Zhang et al. [138] proposed a defect identification model based on machine learning, where they automatically classified the reported alarms into true defects and false positives. The authors designed a set of novel features at variable level, called variable characteristics, for building the classification model and selected 13 base classifiers and two ensemble learning methods for model building. They achieved an 83.36% average accuracy of classification. In [159], the authors used a quantitative model characterizing the impact of illumination with a simple classifier and achieved a best of 94% accuracy in 1865 samples.

### 4.5. Resource Dependency Comparison

The application in a real industrial case scenario requires fast and reliable detection and classification processes. Clearly, reliability is a crucial point since these procedures are stochastic processes with an efficiency that can be improved as the computational cost increases, for example changing the sampling resolution of the system that determines the distance between pixels in digital image. Fine textures require smaller distance between pixels, whereas coarse textures require larger distances. This means that a reduction of resolution (by quantizing the image to fewer levels of intensity) helps to increase the speed of computation, as long as some loss of textural information is acceptable. Although this approach leads to a faster detection process, the success rate can be smaller due to the omission of non-sampled critical features of defects.

Generally speaking, with model-based approaches, the computational complexity is strongly affected by the estimation of stochastic model parameters. Methods such as MRFs, for example, need to be trained before their employment as classifiers. Computational cost and efficiency of a classifier heavily depend on the dimension of the neural network used for the training phase [29,40].

Fractals, instead, are computationally suitable for PC implementation, but have poor accuracy [160]. Statistical approaches using co-occurrence matrices are computationally expensive, thus not suitable for a real-time defect inspection system. However, several studies have widely demonstrated that these are highly accurate techniques [2].

Other statistical approaches, such as LBPs, have a cheap computational cost in real-time applications for texture classification but they have lower performance than co-occurrence matrices and other filtering-based approaches for detecting random textural defects [33].

## 5. Supervised and Non-Supervised Classifiers

The main goals of the visual processes are the detection and classification of defects that can be solved by establishing classifiers. In the previous section, we discuss the approaches that are more related to the low-level image processing level, based on Figure 3. This section compares the methods related to the high-level image processing. Their general goal is to discriminate a specific defect, texture feature, or pattern. Based on their processing mechanics, these classifiers can be classified in two groups: (1) supervised; and (2) non-supervised or semi-supervised classifiers (see Table 3).

Supervised classification methods incorporate the human model—as discussed in Section 3—where the application is searching for features of a predefined class. Detectable features are predefined and the classifier has to be previously trained to recognize them under supervision [40,65,90,103,142,161,162,163]. As part of the supervised classifiers, the *K*-Nearest Neighbor (KNN) classifier is a non-parametric learning algorithm where the output object, classified into classes, uses its local neighborhood to formulate a prediction. The KNN algorithm is among the simplest machine learning algorithms, where *K* is a user-defined constant that defines the number of neighbors to employ for classification. A high *K* value reduces the noise of classification, but makes the boundaries between classes less distinct, thus the best choice must be tuned upon the dataset. In [162,163], Lopez et al. used KNN to classify ceramic tile images based on chromatic features and achieved high performance using high *K*-value, while Mandriota et al. [103] applied KNN to inspect rail surfaces but did not find significant difference in their dataset performance because of the higher *K*-value. There are also numerous good classifier-based implementations, e.g., Wiltschi et al. [90] and Latif-Amet et al. [25] classified images based on the parametric distance. Chan and Pang [73] classified defects by simulating their main features to describe a classifier. Pernkopf [161] used KNN to classify steel surfaces based on dispersions extracted from hidden Markov random fields.

Artificial neural networks are commonly used classifiers due to them being universal function approximators [206]. They are computing systems inspired by biological neural networks that can learn from data and store the knowledge of the classification. The key feature of the neural networks is the iterative learning process in which teaching-samples are presented to the network in batches or minibatches and the weighted connections between neurons are adjusted by the input values associated with the activation function. A Feed-Forward Neural Network (FFNN), described in [40] by Kumar, was applied to classify extracted texture features of textile images and to solve segmentation problem. Monadjemi et al. [65] established a Back Propagation (BP) Neural Network (NN) combined with lower level processes (e.g., co-occurrence matrices, LBP, Gabor filters, etc.) and outperformed a KNN in ceramic texture features classification. Stojanovic et al. [164] used a three-layer BP NN to detect fabric defects with 86.2% success. Within the last decades, BPNNs have been commonly used and successful methods for defect detection: as reported in [165,166], Kuo et al. achieved 91.88% on 160 defective images and 94.38% success rates on 240 defective images. Hung and Cheng [167] used BPNNs with fuzzification technique but with unclear success rate. In [168], the authors achieved a 91–100% detection rate on 16-16 samples with BPNNs, while Zhang et al. [169] achieved a 83.4% success rate with a FFNN. Besides the previously mentioned methods, there are numerous studies about applied neural networks, such as studies reported in [6,62,75,78,85,99,109,170]. In 2012, Cord and Chambon [59] proposed an automatic defect detection method by textural pattern recognition based on a supervised learning method, called AdaBoost, and achieved a 91% detection rate with 12.5% false alarm on 6875 samples. In [198], Shipway et al. investigated three methods of modifying the fluorescent penetrant inspection Random Forest (RF) method, based on the individual performance of decision trees within the RF. Their main attempt was to improve the effectiveness of RF at performing automated defect detection.

Other commonly used supervised classifiers have been developed, such as Self-Organizing Maps (SOM) mainly used for clustering, feature mapping and SVM to classify defects based on features. SVMs can be an appropriate alternative to FFNNs, because they are computationally easier to train and do not have local minimum problems. Therefore, many studies [171,193] have been published about the SVM in defect detection and the authors of [31,194] reported SOM methods. The authors of [12,13,142] performed unsupervised clustering SOM with supervised sample mapping. Supervised classification proved its value in the field of visual inspection. However, it strongly depends by the number of samples and training conditions. Accordingly, the training phase of the classifier often takes time and needs a large defect sample database—which is not always available—but it achieves higher success rates with a longer training phase. Recently, in [172], Li et al. proposed a discriminative representation for patterned fabric defect detection and achieved a 95.8% detection rate with 2.5% false alarms on 600 samples. They classified sample images with the Fisher criterion-based stacked denoising autoencoders (FCSDA) and introduced deep learning for the first time in the defect detection field. Tural et al. [192] recently developed a system using various image processing and filtering method (Bilateral filtering, Sobel filtering, thresholding, and morphological closing) in a combination with SVM to detect and classify defects on bullet shells. They achieved 96% accuracy in a real-time production environment.

The main feature for non-supervised classifiers is the capability of detecting every feature that is not part of the texture and pattern. They represent the other approach of the human detection model, in which the detector is trained with normal samples and every deviation is considered as abnormal. This approach is particularly useful when the spatial distribution of the abnormalities is needed. These methods usually exploit distance-based or thresholding rules to discriminate questionable features. Markou and Singh [196,197] published studies on the novelty for detection approaches using statistical and neural-network-based techniques. During visual inspections, statistical parametric approaches are often used [65,86,89,90,91,92,103]. The essential hypothesis is the Gaussian natural distribution of the data. Gururajan et al. [203] proposed a Gaussian mixture model with Expectation-Maximization features to detect one specific kind of defect, and achieved 93% true positive and 95% true negative detection success rates for six types of soils under four categories of laundering treatments. Zhang et al. [204] combined Gabor transformation with a Gaussian mixture model for plain fabric defect detection, with 87% classification success rate achieved. In [133], the authors proposed two different mixture models to measure pattern likelihoods by using simple parametric thresholding, automatically determined from training data with a 92.67% overall accuracy. Recently, Zhu et al. [115] applied Gabor filter as a pre-process method to reduce the complexity of the fabric signal, and built the over-complete basis set via sparse coding. They achieved a 93.7% defect detection success rate with 9.6% false alarms on 284 samples. Susan and Sharma [207] proposed a new unsupervised automated texture defect detection method that uses a Gaussian mixture entropy model to determine the optimal window size for feature extraction. Recently, Mei et al. [205] developed an unsupervised learning based method by using only defect free samples for model training. The approach was carried out by reconstructing image patches with convolutional denoising autoencoder networks at different Gaussian pyramid levels, and synthesizing detection results from these different resolution channels.

### Deep Learning for Defect Detection

Deep learning is one of fastest growing fields in computer sciences, due to its ability to solve highly complex problems [208]. The rich accumulation of traditional machine learning techniques resulted in the evolution of deep learning that also gained its inspiration from statistical learning. Most of the approaches mentioned in the previous sections are considered as traditional solutions, where the focus is on the explicitly engineered features which can be challenging to describe in complex cases. However, deep learning uses data representation learning to perform tasks, that transform data into complex, abstract representations that enable the features to be learnen for systems (e.g., feature learning). This ability of deep learning overcomes the requirement of complex features for a specific defect. Both deep learning and traditional machine learning are data-driven artificial intelligence techniques able to successfully model deterministic rules, which are often incomprehensible to humans and relationships between input and output. Moreover, deep learning disposes the capability of performing feature learning, model construction and model training, by selecting different kernels or tuning and optimizing parameters.

In 2018, Wang et al. [209] summarized the capabilities of deep learning for smart manufacturing and highlighted how deep learning changes future trends in industry. Within the past years, a number of relevant studies have been published on defect detection solutions using deep learning [175,210,211,212,213,214]. Lin, Hui et al. [214] developed a CNN called LEDNet to detect and classify defect on LED chips, where they achieved relatively low inaccuracy of 5.05%. Sun et al. [180] compared back-propagation neural networks and learning vector quantization performance in detecting the four commonly seen bur defect on thermal fuses. In 2015, Ren et al. [215] introduced a method by combining the region proposal network (RPN) and Faster Region-based Convolutional Neural Network (Faster R-CNN) for object detection to generate nearly cost-free region proposals. In [216], the authors used a Faster R-CNN-based visual inspection method to detect and classify five defect types with 90.6%, 83.4%, 82.1%, 98.1%, and 84.7% average precisions. Notably, their method performed the task significantly more quickly than a traditional CNN based method, which is necessary for real-time implementation. Wang et al., [173] developed a faster R-CNN algorithm to solve the speed problem of CNNs and to locate small defects in geometrically complex products where they achieved 72% detection and 81% classification accuracy. Liu et al. [174] introduced a defect detection method based on semantic segmentation. For this, they used a development and extension of CNN called Fully Connected Networks (FCN) where they transformed the fully connected layer of a CNN into a convolution layer. They achieved 99.6% accuracy on the German DAGM 2007 dataset. Recently, Kumar et al. [178] used a deep convolutional neural network (DCNN) to detect and classify defect in sewers and achieved and average of 86.2% testing accuracy, 87.7% precision and 90.6% recall. Later, Brackenbury et al. [179] compared three different classification strategies for DCNNs to detect structural faults in masonry arch bridges. Their study shows the the importance of the right structure and method choice based on the dataset.

Li et al. [176] combined Gabor filters and Pulse Coupled Neural Network (PCNN) for fabric defect detection and achieved 98.6% accuracy. The PCNN model was developed by Johnson et al. [217]. It was inspired by the study in [218] by Echorn about the synchronous dynamics of neuronal activity in cat visual cortex. Chen et al. [177] stated that, recently, PCNN models are the most potential method in image processing due to its high potential by resolving the problem of parameter estimation of segmentation problems.

In 2018, Sacco et al. [181] developed a CNN based system for automatic quality control for fiber placement manufacturing, however they failed to achieve satisfactory results due to their small (only 200 samples/defect) training dataset that led them to over-fit their network. This factor is one of the most crucial factor by employing CNNs. To solve this common problem, Yang et al. [219] developed a promising and robust method as virtual defect rendering, that can solve the problem of small datasets. In a recent study [182], Yang et al. developed a DCNN based system to detect and classify defects that can occur during laser welding in battery manufacturing. Besides that, they proposed a novel model called Visual Geometry Group (VGG) model to improve the efficiency of defect classification. Their test on 8000 samples with a 99.87% accuracy proved that the pre-trained VGG model has small model size, lower fault positive rate and shorter training time and prediction time. It is notable that their model is highly suitable for quality inspection in an industrial environment. Following the evolution of the industrial quality control field, there is an unequivocal need for general solutions to solve complex challenges that can be served by deep learning.

## 6. Conclusions and Future Directions

This paper provides a review of defect detection methodologies described in more than 220 scientific contributions. A significant amount of works is based on statistical observations and uses statistical or filter-based methods. The Gabor filter is one of the most commonly used methods. However, most of the studies present specific limitations, being heavily dependent on the pattern, material and texture. Solving the segmentation and windowing problems of overlapping objects is still a ponderous topic approached by several researchers. Images having color features can multiply the complexity of these problems.

Neural networks are a powerful technique often employed in artificial image processing since they can nearly solve every classification problem. However, the main drawback is the required large amount of training samples. In artificial image processing, this issue can be easily solved with labeled datasets, or applying stochastic solutions (i.e., mini-batches). However, in other fields such as robotics, or other systems that learn from real-world operations, it is still a challenging problem. Improving the training efficiency and convergence capability of neural networks is an ongoing research area. It is also notable that large neural networks used for deep learning require significant computational resources, which lead to an unavoidable parallelization of the challenges [220].

Supervised learning methods are well-functioning and straightforward to use. Due to their capabilities, supervised methods are the most preferred for classification in industry but in many cases they are time consuming to train and require large datasets.

Unsupervised learning is used for density estimation, dimensionality reduction and clustering problems. However, in many cases, unsupervised methods have shown lower efficiencies than supervised learning methods. Natural supervision is an emerging topic in the field, due to its similarities to biological learning behaviors. From another perspective, artificial neural networks are inspired by biological neural networks, but do not necessarily replicate them. Back propagation is the essence to train many artificial neural networks, although no such mechanism exactly exists in biological networks [221]. This means that biological neural networks gave a good inspiration to develop artificial neural networks that can be used as classifiers; however, sufficiently modeling them for technological use is still an unsolved topic.

In artificial image processing, different textural databases are available for testing, although several studies do not provide satisfactory results due to the lack of testing samples and frequent inconsistency of such databases. Moreover, there is still a huge demand for developing general defect detection methods able to deal with any kind of defect on every kind of material, and also able to establish a general and reliable defect description system. Due to the lack of solutions, there is a huge demand in industry to increase the defect identification efficiency with multi-sensory systems applications. To this aim, deep learning is the emerging field that could solve the generalization requirement and hyper-complexity of problems without drastically increasing computational costs.

## Figures and Tables

**Table 1:** Strengths and weaknesses of different image processing techniques.

Approach	Method	Strengths	Weaknesses
Statistical	Histogram properties	Simplicity, invariant to translation and rotation.	Requires the assumption that the intensity of defective regions are separable.
	Co-occurrence matrix	The spatial relation of extracted image pixels is complete and accurate.	Computation and memory requirements are relatively high.
	Local binary pattern	Gray invariance, can quickly extract discriminative features with rotation.	Highly sensitive to noise, scale change.
	Other gray level statistics	Suitable for low resolution images.	Low timeliness and no automatic threshold selection.
	Auto-correlation	Easy to use on textures that are repetitive in nature, such as textiles.	Unsuitable for random textures with irregularly arranged textural elements.
	Registration-based	Can mix different sensory images or acquired from different views or at different times.	Heavily depends on the keypoint detection algorithms or the similarity measurement strategies.
Statistical	Primitive measurement	Simple, easy to understand and implement.	Sensitive to non-linear noises.
	Edge features	Easy to realize and capable to extract some low-order features of the image.	Susceptible to noise and only suitable for images with low resolution.
	Skeleton representation	Built up from multiple statistical histogram analysis as a structure. Mixes their strengths.	Heavily depends on the applied statistical methods’ weaknesses.
	Morphological operations	Computationally simple and highly suitable for random or natural textures.	Only suitable for aperiodic defects.
Filter	Spatial domain filtering	Spatial information extraction, possible to use for localization.	Computation and memory requirements scale heavily. Translation, expansion and rotation dependent.
	Frequency domain analysis	Invariant to translation, expansion and rotation.	Difficult to realize non-interference when dealing with frequency-domain components related to background or defect.
	Joint spatial/spatial frequency	Can outperform space or frequency invariant based methods when the signal and noise overlap in both space and frequency domains.	High complexity. Hard to determine the optimal filter parameters and no rotation invariance.
Model based	Fractal model	The overall information of images can be expressed by partial features.	Detection accuracy is unsatisfactory and have limitation on images without self-similar.
	Random field model	Can be combined with statistical and spectral methods for segmentation applications to capture the local texture orientation information.	Cannot detect small defects. Not suitable for global texture analysis. Strong spatial constraint.
	Texem model	Potentially useful for image segmentation problems even on colored images.	Requires significantly large training dataset to train the model.
	Auto-regressive	High performance for texture related problems.	Tend to be limited to low-resolution images since memory and computation requirements grow with the size of the image.

**Table 2:** A selection of most commonly used textural defect detection methods.

Approach	Method	References
Statistical	Histogram properties	[6,7,8,9,10,11,12,13,14]
	Co-occurrence matrix	[6,11,15,16,17,18,19,20,21,22,23,24,25,26,27,28,29,30]
	Local binary pattern	[12,28,30,31,32,33,34,35,36,37,38]
	Other gray level statistics	[21,23,39,40,41,42,43,44]
	Auto-correlation	[23,45,46]
	Registration-based	[47,48,49,50]
Structural	Primitive measurement	[51,52]
	Edge features	[53]
	Skeleton representation	[54,55]
	Morphological operations	[51,52,56,57,58,59,60]
Filter based	Spatial domain filtering	[26,61,62,63,64,65,66,67,68,69,70]
	Frequency domain analysis	[22,71,72,73,74,75,76,77,78,79,80,81,82,83,84]
	Joint spatial/spatial frequency	[25,33,39,68,85,86,87,88,89,90,91,92,93,94,95,96,97,98,99,100,101,102,103,104,105,106,107,108,109,110,111,112,113,114,115,116]
Model based	Fractal model	[117,118,119,120]
	Random field model	[26,121,122,123,124,125,126,127,128,129,130,131,132]
	Texem model	[133]
	Auto-regressive	[24,37,134,135,136,137,138,139,140,141]
Color texture analysis for defect detection		[36,43,52,69,94,133,142]

**Table 3:** The taxonomy and a supplementary list of references about supervised and unsupervised or semi-supervised classifiers used for defect detection.

Approach	Method	References
Supervised classifiers	*K*-nearest neighbor	[25,73,90,103,161,162,163]
	NNs & Deep learning	[6,29,40,59,62,65,75,78,85,99,109,164,165,166,167,168,169,170,171,172,173,174,175,176,177,178,179,180,181,182,183,184,185,186,187,188,189,190,191]
	SOM and SVM	[12,13,21,30,31,38,142,148,149,171,192,193,194,195]
Unsupervised/semi-	Statistical/Novelty detection	[58,65,86,89,90,91,92,103,115,129,196,197,198,199,200,201,202]
supervised classifiers	Gaussian mixture model	[80,203,204,205]
